# Structural insight into substrate and product binding in an archaeal mevalonate kinase

**DOI:** 10.1371/journal.pone.0208419

**Published:** 2018-12-06

**Authors:** Bradley R. Miller, Yan Kung

**Affiliations:** Department of Chemistry, Bryn Mawr College, Bryn Mawr, PA, United States of America; University of Queensland, AUSTRALIA

## Abstract

Mevalonate kinase (MK) is a key enzyme of the mevalonate pathway, which produces the biosynthetic precursors for steroids, including cholesterol, and isoprenoids, the largest class of natural products. Currently available crystal structures of MK from different organisms depict the enzyme in its unbound, substrate-bound, and inhibitor-bound forms; however, until now no structure has yet been determined of MK bound to its product, 5-phosphomevalonate. Here, we present crystal structures of mevalonate-bound and 5-phosphomevalonate-bound MK from *Methanosarcina mazei* (MmMK), a methanogenic archaeon. In contrast to the prior structure of a eukaryotic MK bound with mevalonate, we find a striking lack of direct interactions between this archaeal MK and its substrate. Further, these two MmMK structures join the prior structure of the apoenzyme to complete the first suite of structural snapshots that depict unbound, substrate-bound, and product-bound forms of the same MK. With this collection of structures, we now provide additional insight into the catalytic mechanism of this biologically essential enzyme.

## Introduction

The mevalonate pathway is responsible for the biosynthesis of steroids and isoprenoids, also known as terpenes and terpenoids, the largest and most structurally diverse class of natural products. Distributed widely across eukaryotes, bacteria, and archaea, the mevalonate pathway consists of seven enzymes that convert three molecules of acetyl-CoA to isopentenyl pyrophosphate (IPP) and dimethylallyl pyrophosphate (DMAPP), the isomeric, five-carbon precursors to all steroids and isoprenoids [1]. Recently, the biological production of isoprenoids and their derivatives has been enabled by the expression of mevalonate pathway enzymes in engineered host microorganisms [2–6], leading to the possibility of widespread industrial biosynthesis of useful isoprenoid drug and biofuel compounds. In addition, enzymes of the mevalonate pathway are currently being used and explored as targets for antimicrobial and anticancer drugs and may also be enzyme targets to combat neurodegenerative diseases [7–9].

A key regulatory step in the mevalonate pathway is the ATP-dependent phosphorylation of (*R*)-mevalonate to (*R*)-5-phosphomevalonate catalyzed by the enzyme mevalonate kinase (MK), a member of the galacto-/homoserine/mevalonate/phosphomevalonate (GHMP) kinase family [10]. Currently available crystal structures of MK show that the enzyme has two domains: ATP binds an N-terminal domain, as seen in the structure of ATP-bound mammalian MK from *Rattus norvegicus* (RnMK, PDB entry 1KVK) [11], while mevalonate binds within a cavity between the N- and C-terminal domains, as seen in the structure of mevalonate-bound MK from the eukaryotic parasite *Leishmania major* (LmMK, PDB entry 2HFU) [12]. Until now, these mevalonate- and ATP-bound eukaryotic MK structures comprise the only known structures of substrate-bound MK.

These structural data in combination with mutational studies have led to a mechanistic proposal for MK catalysis. Here, biochemical and inhibition studies have indicated that in some organisms substrate-binding is ordered, where mevalonate binds the enzyme before ATP [13, 14]. However, as described above crystal structures of MK have been determined with only mevalonate or ATP bound, suggesting a random order where either substrate may bind first. Regardless, upon binding both substrates it has been proposed that a conserved aspartate side chain deprotonates the C5-hydroxyl group of mevalonate [1, 12]. Mutation of this aspartate in human MK to alanine or asparagine led to a dramatic decrease in activity (>40,000-fold decrease in *k*_cat_) with no significant difference in mevalonate or ATP binding, with *K*_m_ and *K*_d_ values left virtually unchanged [15]. These results suggested that this aspartate residue serves a catalytic role in deprotonating the C5-hydroxyl group of mevalonate for attack on the ATP γ-phosphate. However, the mevalonate-bound LmMK structure shows that the aspartate side chain carboxylate is located >5 Å from the mevalonate C5-hydroxyl group [12]. In addition, a conserved lysine residue interacts with the catalytic aspartate, and mutation of this lysine to methionine in the human enzyme resulted in a ~56-fold decrease in *k*_cat_ and an increase in *K*_d_ of about 16- and 56-fold for mevalonate and ATP, respectively, suggesting an important, though not essential, role in substrate binding and catalysis [16]. Indeed, the crystal structure of ATP-bound RnMK shows this lysine making interactions with both the γ-phosphate group of ATP as well as the catalytic aspartate [11]. Following deprotonation of the mevalonate C5-hydroxyl group, the resulting alkoxide nucleophile may then attack the γ-phosphate of ATP. Phosphoryl transfer yields ADP and the product, 5-phosphomevalonate.

In addition to the mevalonate- and ATP-bound structures of eukaryotic LmMK and RnMK, respectively, several crystal structures of MK from other organisms are known, including the apo structure of MK from the archaeal organism *Methanosarcina mazei* (MmMK), without any ligands bound (PDB entry 4HAC). The activity of MmMK has been previously studied, illustrating Michaelis-Menten kinetic behavior that is consistent with other MK homologs [17], with *K*_m_ values for mevalonate and ATP of 68 μM and 464 μM, respectively. However, corresponding crystal structures of ligand-bound MmMK have not yet been determined, and no structure of a 5-phosphomevalonate, product-bound MK has been solved until now.

To shed greater light onto the mechanism of MK and to further investigate archaeal MK in particular, we determined the X-ray structures of MmMK bound with the substrate mevalonate and with the product 5-phosphomevalonate. Interestingly, we found remarkably few direct interactions between archaeal MmMK and mevalonate, none of them involving side chains, an observation that stands in contrast to the structure of mevalonate-bound LmMK [12], until now the only structure of MK bound with mevalonate, which depicts several direct contacts between the enzyme and its substrate. We also compare our mevalonate-bound MmMK structure with the structure of apo-MmMK, illustrating only a slight conformational tightening that occurs upon substrate binding. Finally, both structures of mevalonate- and 5-phosphomevalonate-bound MmMK have offered additional new insight into the catalytic mechanism. Together, our substrate- and product-bound MmMK structures provide a more complete view of ligand binding and catalysis in this metabolically important enzyme.

## Materials and methods

### Cloning, expression, and purification

A codon-optimized gene encoding MmMK was cloned using NdeI and BamHI restriction enzymes into pSKB3, a modified pET28b vector (Novagen) that includes an N-terminal, TEV-protease-cleavable hexahistidine tag and a kanamycin resistance cassette. The resulting plasmid was transformed into *Escherichia coli* DH10B cells (Invitrogen), its sequence was confirmed (Quintara Biosciences), and it was then transformed into BL21(DE3) cells (Invitrogen) for protein expression.

All chemicals were purchased from Sigma-Aldrich, unless otherwise indicated. Cells were grown in lysogeny broth (ThermoFisher) supplemented with 50 μg/mL kanamycin at 37°C until the optical density at 600 nm reached ~0.6. Protein expression was induced with 0.5 mM isopropyl β-d-1-thiogalactopyranoside (IPTG) and proceeded for 18 hours at 16°C. Cells were harvested by centrifugation at 5,000 × *g* for 10 min, flash frozen in liquid nitrogen, and stored at -80°C until use. Cells were resuspended in lysis buffer (50 mM Tris pH 7.7, 200 mM NaCl, 10% glycerol, 10 mM imidazole) with 0.5 U/μL benzonase (Millipore) and 0.5 mM phenylmethanesulfonyl fluoride (PMSF) and lysed by sonication on ice at 40% amplitude for 9 min with three-second bursts and five-second rests. The lysate was clarified by centrifugation at maximum speed (~37,000 × *g*) for 30 min at 4°C, and the supernatant was applied to a Ni-NTA column equilibrated with lysis buffer. MmMK was eluted from the column in fractions using lysis buffer with 300 mM imidazole at 4°C and assessed for purity by SDS-PAGE. Fractions containing the highest purity were pooled, and the sample was dialyzed overnight at 4°C against the storage buffer, which contained 20 mM Tris pH 7.7, 10 mM MgCl_2_, and 10% glycerol. Purified MmMK was concentrated to 11 mg/mL, flash frozen dropwise in liquid nitrogen, and stored at -80°C.

### Protein crystallization

Crystals of MmMK have been grown previously [18], and a resulting apo-MmMK structure was deposited in the Protein Data Bank (PDB entry 4HAC). However, because we were not able to reproduce crystals from these published conditions, we identified new crystallization conditions for mevalonate-bound MmMK via sparse-matrix screening by sitting-drop vapor diffusion using a Crystal Gryphon (Art Robbins Instruments) with 11 mg/mL MmMK in storage buffer. Crystals were observed in 100 mM Bis-Tris pH 5.5, 200 mM sodium potassium tartrate, and 30% polyethylene glycol (PEG) 3,350. Crystallization conditions then were optimized by hanging-drop vapor diffusion with varying sodium potassium tartrate, PEG molecular weight, and PEG concentrations. The best crystals grew overnight using 15–25% PEG 8,000 or 10,000. Crystals were then looped, washed in mother liquor, crushed via vortexing, and used as seeds for microseeding. Seeded drops contained 1.0 μL of the MmMK sample with 1 mM (*R*)-mevalonate, 5 mM AMPPNP, and 5 mM MgCl_2_, 0.8 μL crystallization solution, and 0.2 μL of the seed stock. Diffraction-quality plate crystals grew in crystallization solutions that contained 100 mM Bis-Tris pH 5.5, 200–300 mM sodium potassium tartrate, and 15–25% PEG 8,000 or 10,000. Crystals were cryoprotected using 100 mM Bis-Tris pH 5.5, 20% PEG 8,000 or 10,000, 20% glycerol, 1 mM (*R*)-mevalonate, 5 mM AMPPNP, and 5 mM MgCl_2_ before flash-cooling in liquid nitrogen. Despite the presence of AMPPNP at 5 mM concentrations, the resulting structures did not yield any electron density in the ATP binding site, similar to prior crystallographic studies on LmMK [12].

Crystals of MmMK with (*R*)-5-phosphomevalonate were grown and optimized using the same crystallization solution as with (*R*)-mevalonate-bound MmMK, with 1 mM (*R*)-5-phosphomevalonate and 5 mM MgCl_2_.

### X-ray data collection, structure determination, and refinement

X-ray diffraction data to 2.10 Å and 2.46 Å resolution for mevalonate-bound and 5-phosphomevalonate-bound MmMK crystals, respectively, were collected at the Advanced Photon Source (APS) beamline 24-ID-E. Both data sets were indexed, merged, and scaled using iMOSFLM [19] in the space group *P*2_1_2_1_2_1_ with two molecules in the asymmetric unit. The structures were solved by molecular replacement using Phaser [20] in the Phenix suite [21] using one protein monomer from the apo-MmMK structure (PDB entry 4HAC) as the search model. The structures were refined using phenix.refine [22] with model building in Coot [23]. Complete X-ray collection and refinement statistics are provided in Table 1.

**Table 1:** X-ray data collection and refinement statistics.

	MmMK + mevalonate	MmMK + 5-phosphomevalonate
PDB entry	6MDE	6MDF
**Diffraction Data**		
Beamline	APS, 24-ID-E	APS, 24-ID-E
Wavelength (Å)	0.9792	0.9792
Space group	*P*2_1_2_1_2_1_	*P*2_1_2_1_2_1_
Unit cell		
*a*, *b*, *c* (Å)	54.05, 59.33, 191.10	53.97, 58.58, 190.03
Resolution (Å)	52.01–2.10 (2.18–2.10)	51.92–2.46 (2.55–2.46)
Wilson B (Å^2^)	28.16	39.55
Total reflections	126,622 (12,658)	95,987 (8,496)
Unique reflections	36,591 (3,601)	22,260 (2,135)
Multiplicity	3.5 (3.5)	4.3 (4.0)
Completeness (%)	99.14 (99.39)	96.95 (96.30)
Mean *I*/*σ*(*I*)	9.97 (2.10)	8.73 (2.85)
*R*_merge_	0.1107 (0.9636)	0.1031 (0.3752)
*R*_meas_	0.1302 (1.131)	0.1171 (0.4303)
CC1/2	0.995 (0.6)	0.995 (0.843)
CC*	0.999 (0.866)	0.999 (0.957)
**Refinement**		
*R*_work_	0.1955 (0.2913)	0.2003 (0.2576)
*R*_free_	0.2496 (0.3536)	0.2672 (0.3447)
No. protein/ligand atoms	4,839	4,659
r.m.s.d. bonds (Å)	0.008	0.003
r.m.s.d. angles (°)	0.883	0.510
Avg. B factor (Å^2^)	38.53	48.88
Ramachandran analysis		
Favored (%)	96.33	96.00
Allowed (%)	3.67	4.00
Outliers (%)	0.00	0.00
MolProbity Clashscore	8.21	5.12

Statistics for highest resolution shell shown in parentheses

## Results and discussion

### Overall structure

**Fig 1:**
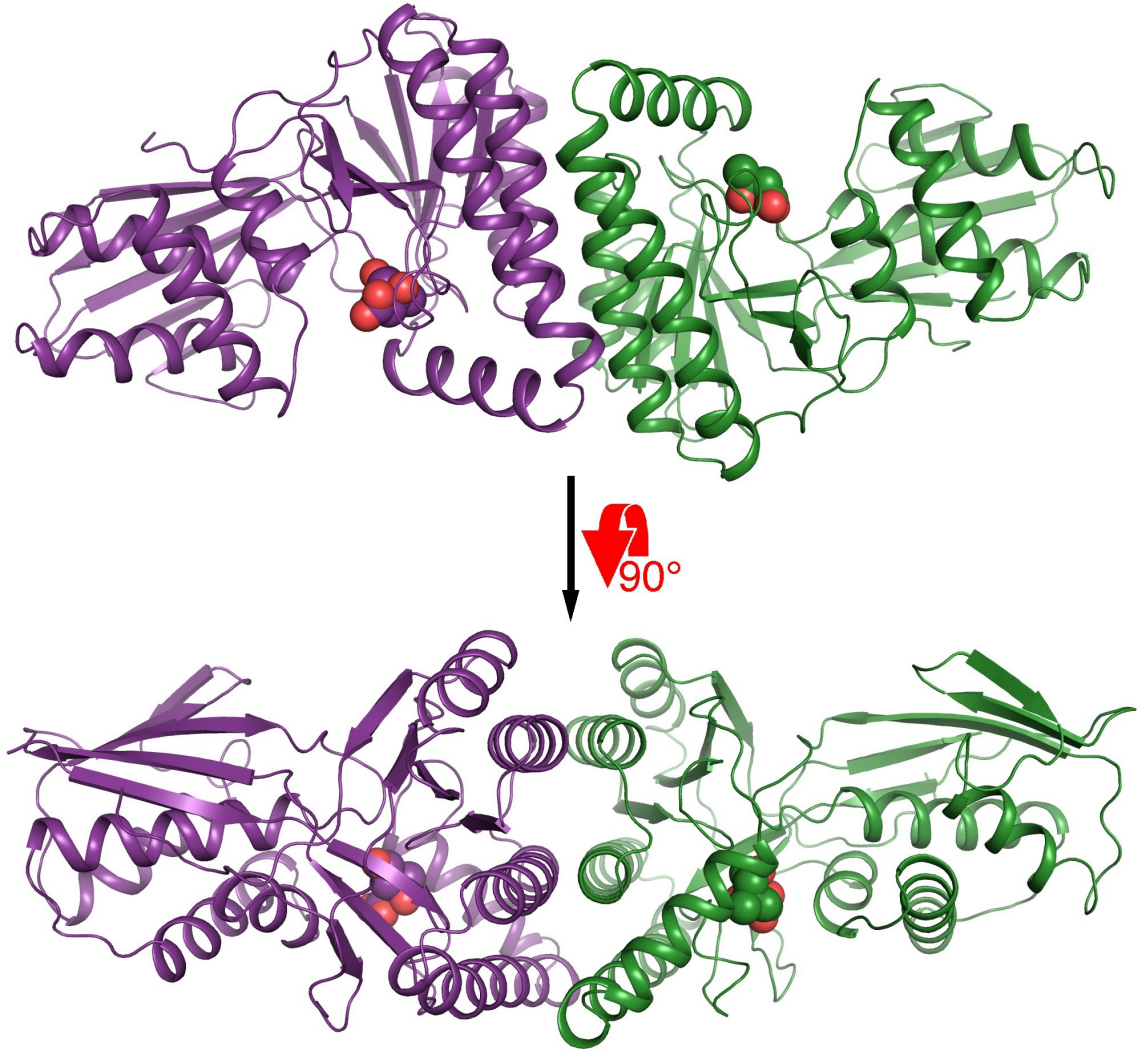
Overall structure of homodimeric mevalonate-bound MmMK. Protein is shown in cartoon, with monomers colored in violet and green, and mevalonate is shown in spheres.

MmMK bound with mevalonate or 5-phosphomevalonate crystallized in the *P*2_1_2_1_2_1_ space group with two molecules in the asymmetric unit, forming a homodimeric structure (Fig 1). For both structures, electron density was stronger for chain A than for chain B, likely due to the latter’s relative lack of crystal lattice contacts. Although there are examples of MKs that have crystallized as monomers, such as MK from *Methanocaldococcus jannaschii* (MjMK, PDB entries 1KKH and 1VIS) [24, 25], most prior MK structures have depicted homodimers, including RnMK (PDB entries 1KVK and 2R32) [11, 26], LmMK (PDB entries 2HFS and 2HFU) [12], human MK (PDB entry 2R3V) [26], and MmMK (PDB entry 4HAC), consistent with solution data [15, 16]. In all homodimeric cases, MK monomers are arranged side-by-side with two-fold symmetry such that the two active sites lie on opposite sides of the homodimer. The dimeric interface is composed of three α-helices from each monomer, representing a buried surface area of ~1200 Å^2^, as calculated in PISA [27].

**Fig 2:**
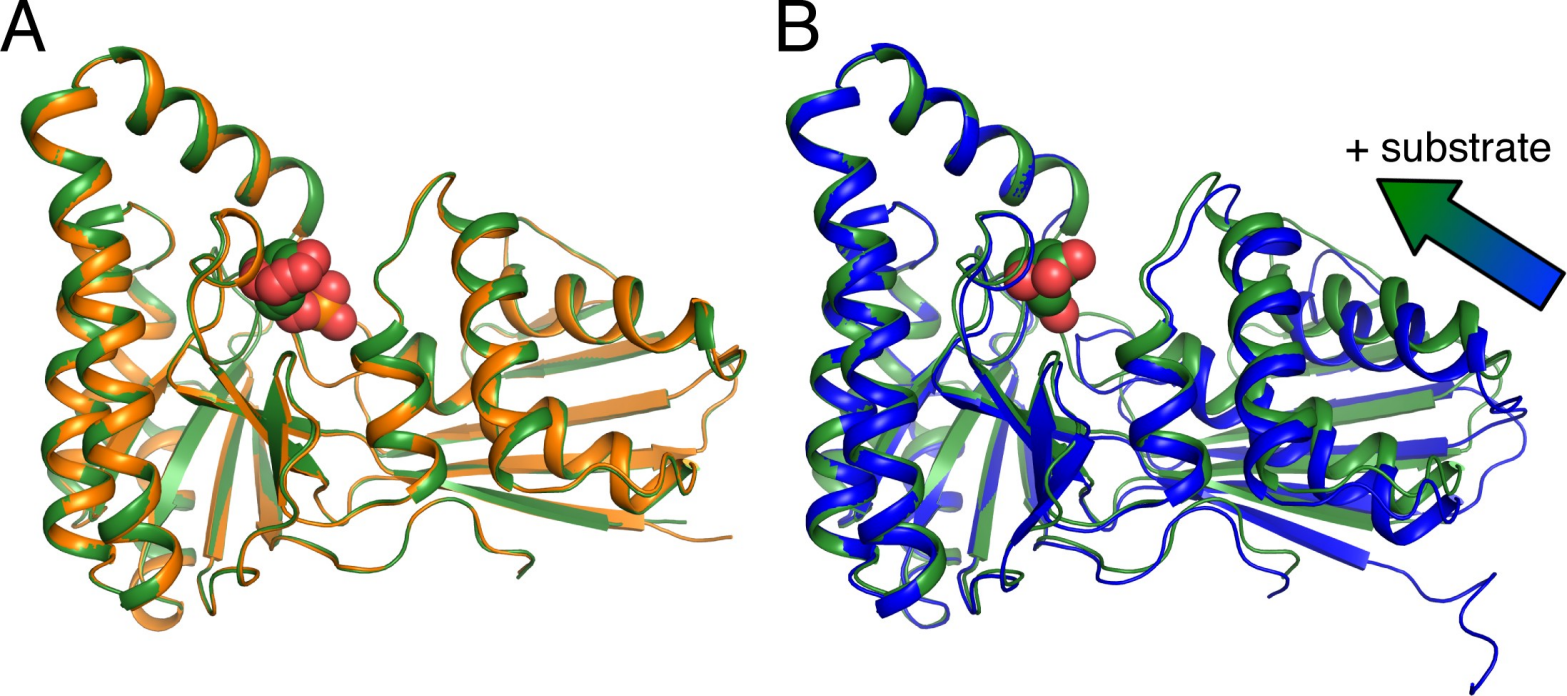
Superposition of monomers from MmMK structures. (A) Structures of mevalonate-bound MmMK (green) and 5-phosphomevalonate-bound MmMK (orange), with ligands as spheres, are virtually superimposable, with no significant structural differences. (B) Structures of apo-MmMK (blue, PDB entry 4HAC) and mevalonate-bound MmMK (green), with mevalonate shown as spheres, illustrate a slight conformational tightening upon substrate binding (arrow).

Our structures of mevalonate- and 5-phosphomevalonate-bound MmMK align very well with each other, with root-mean-square deviation for C_α_ atoms (rmsd) of 0.18 Å, indicating no significant structural differences between the substrate- and the product-bound enzyme (Fig 2A). Alignment of these structures with the prior structure of apo-MmMK yields a somewhat greater rmsd of 0.45–0.71 Å, and a superposition of monomers from the apo-MmMK and mevalonate-bound MmMK structures shows a slight contraction upon ligand binding (Fig 2B). Here, the C-terminal region exhibits a small, pivoted motion toward mevalonate, which closes and tightens the overall structure. Besides this modest adjustment, there are no other significant structural movements upon ligand binding.

### Substrate-binding site

**Fig 3:**
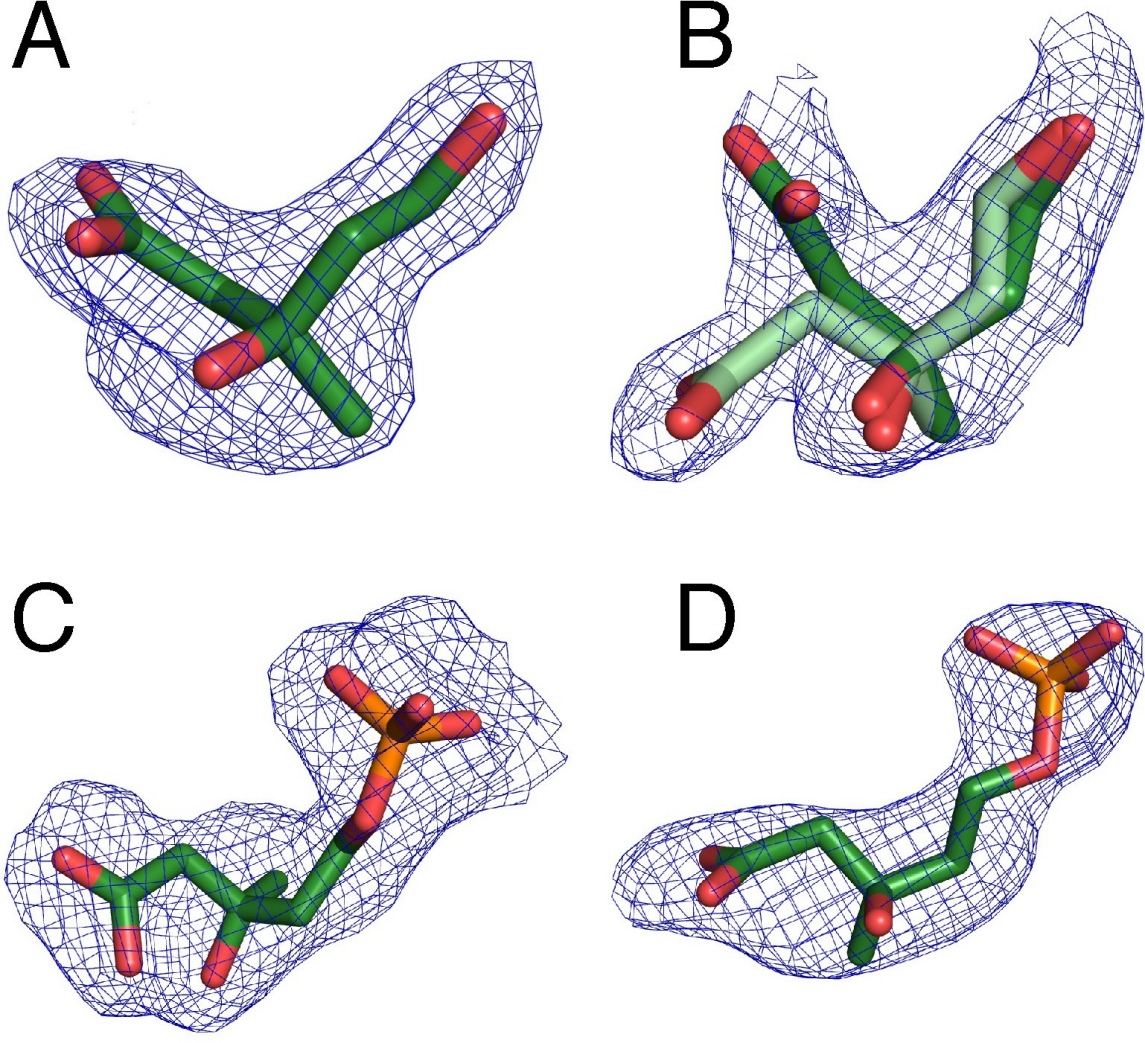
Omit electron density for mevalonate and 5-phosphomevalonate. Mevalonate in (A) chain A and (B) chain B (with alternate conformations in light and dark green) in the structure of substrate-bound MmMK. 5-phosphomevalonate in (C) chain A and (D) chain B in the structure of product-bound MmMK. Polder *mF*_*o*_*−DF*_c_ omit density calculated in Phenix [28] and contoured at 3.0 σ is shown in blue mesh.

As described above, each MK monomer consists of two domains, where the active site lies on a surface cleft at the interdomain interface. Positive difference maps in the mevalonate and the 5-phosphomevalonate MmMK data showed electron density in the substrate-binding sites of both monomers in the asymmetric unit, representing the binding of substrate and product, respectively (Fig 3A–3D). In both structures, electron density for the bound ligands was clearer in chain A than in chain B, a feature that was also observed for the protein chains, as described above. Electron density also indicated that two conformations of mevalonate were present in chain B of the mevalonate-bound structure (Fig 3B), and occupancy refinement in Phenix [21] yielded occupancies of ~0.5 for both conformers. These two mevalonate conformations differ only in the position of the carboxylate group, which faces the solvent exterior, indicating that this moiety may “swing” into two different orientations at the protein surface. The rest of the mevalonate molecule, including the reactive C5-hydroxyl group, remains in the same position across both conformers, suggesting that bound mevalonate with either carboxylate-group conformation would be competent for catalysis.

**Fig 4:**
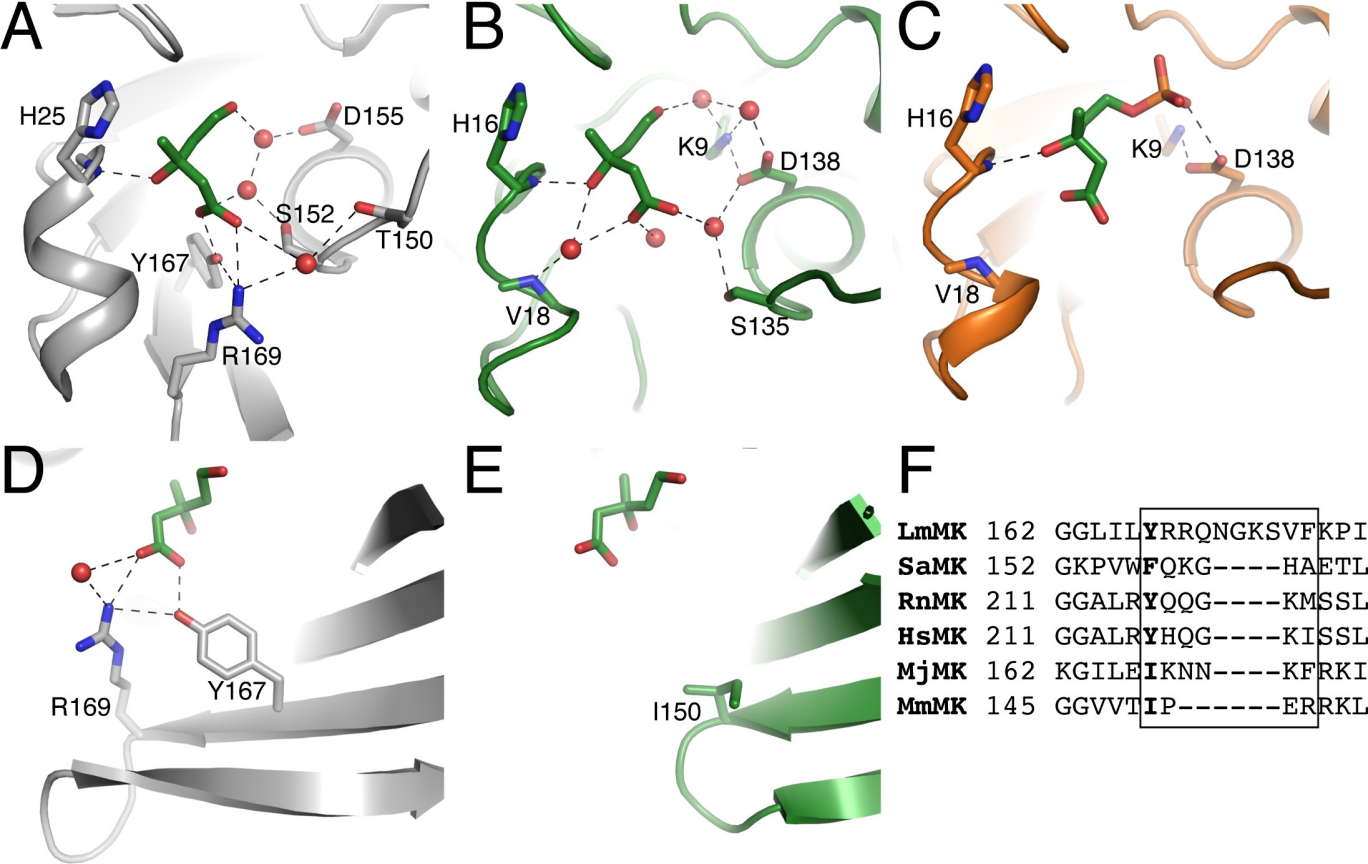
Comparison of LmMK and MmMK mevalonate-binding sites. (A) Structure of LmMK (grey) with mevalonate bound (green sticks). (B) Structure of chain A of MmMK (green) with mevalonate bound (green sticks). (C) Structure of MmMK (orange) with 5-phosphomevalonate bound (green sticks). (D) Residues on a β-strand in LmMK directly interact with the carboxylate group of mevalonate, while (E) the corresponding β-strand of MmMK is truncated and thus cannot interact with mevalonate. Residues shown in sticks, water molecules shown as red spheres, and hydrogen-bonding interactions shown as dashed lines. (F) Sequence alignment of MK homologs, depicting the β-sheet region near mevalonate in the LmMK structure (boxed) and the residue corresponding to the Tyr167 of LmMK that interacts with mevalonate in bold.

Mevalonate binds to MmMK at the same location and in a similar position as previously observed in the structure of mevalonate-bound LmMK [12]. However, in both chains of the structure mevalonate makes very few direct contacts with the MmMK protein, in contrast with the several contacts between mevalonate and LmMK. In the LmMK structure (Fig 4A), the mevalonate carboxylate group interacts directly with Tyr167 and Arg169, which are from the same β-strand (Fig 4A and 4D) Meanwhile, the mevalonate C3-hydroxyl group forms a hydrogen bond with the backbone amide of His25, whose side chain also abuts the mevalonate C3-methyl group. This dual role of conserved His25 was previously proposed to enable the enzyme to select the (*R*)-enantiomer of mevalonate over the (*S*)-enantiomer in all MKs whose stereospecificities have been demonstrated [12]. In addition, water molecules form hydrogen-bond bridges between the substrate and LmMK, including Thr150, Ser152, and Asp155.

However, many of the contacts observed in the LmMK structure are lost in our mevalonate-bound MmMK structure (Fig 4B), where the only direct interaction remaining is between the mevalonate C3-hydroxyl group and His16 (MmMK numbering, corresponding to His25 of LmMK). In fact, the β-strand of LmMK that directly interacts with the mevalonate carboxylate (Fig 4D) is shortened considerably in MmMK such that there are no residues in this region that can reach mevalonate (Fig 4E); in place of the LmMK residue Tyr167 that makes a hydrogen bond with mevalonate, MmMK contains Ile150 (Fig 4E and 4F), which is not capable of interacting with mevalonate. It has been previously noted that this β-strand/loop region appears to be flexible in other MKs and is composed of residues that might also be involved hydrogen-bonding [12]. Indeed, the previously solved structures of eukaryotic RnMK [11, 26], bacterial MK from *Staphylococcus aureus* (SaMK, PDB entry 2X7I) [29] and *Streptococcus pneumoniae* (SpMK, PDB entry 2OI2) [30], and archaeal MjMK [24, 25] all contain Tyr, Gln, Lys, or Asn in this region that may be involved in mevalonate binding (Fig 4F). However, our structure shows that MmMK lacks this region of the β-sheet altogether (Fig 4E), leaving the mevalonate carboxylate group more exposed to the solvent.

Instead of forming direct interactions with the protein, mevalonate bound to MmMK interacts with several ordered water molecules that themselves form hydrogen bonds to the protein (Fig 4B). In this way, water molecules form a cage around the substrate to mediate bridging hydrogen bonding interactions with the protein, including with the backbone amide of Val18 as well as the side chains of Lys9, Ser135, and Asp138. In addition, mevalonate binds slightly differently in both chains of the structure. Clear density was observed for mevalonate in chain A (Fig 3A), but as described above the density in chain B was less clear and indicated two alternate conformations (Fig 3B), each at approximately 0.5 occupancy. Although most of the mevalonate molecule exhibits the same positioning, the carboxylate group is pointed in different directions across both conformations. Because MmMK lacks the β-strand region described above that directly binds the carboxylate group of mevalonate in LmMK, the mevalonate carboxylate apparently remains flexible and may sample different conformations, none of which make direct interactions with the protein.

In the 5-phosphomevalonate-bound MmMK structure (Fig 4C), the mevaldyl moiety of the product is located in a similar position as in the mevalonate-bound MmMK structure, displaying the same lack of direct contacts with the enzyme. The phosphate group, however, interacts with Asp138, a catalytically important residue discussed in greater detail below. In comparing the mevalonate- and phosphomevalonate-bound structures, there are no significant changes in the protein backbone, as noted above, and residues in the active site also do not display significantly altered orientations, indicating that the transformation of the substrate to the product does not trigger major protein rearrangements.

**Fig 5:**
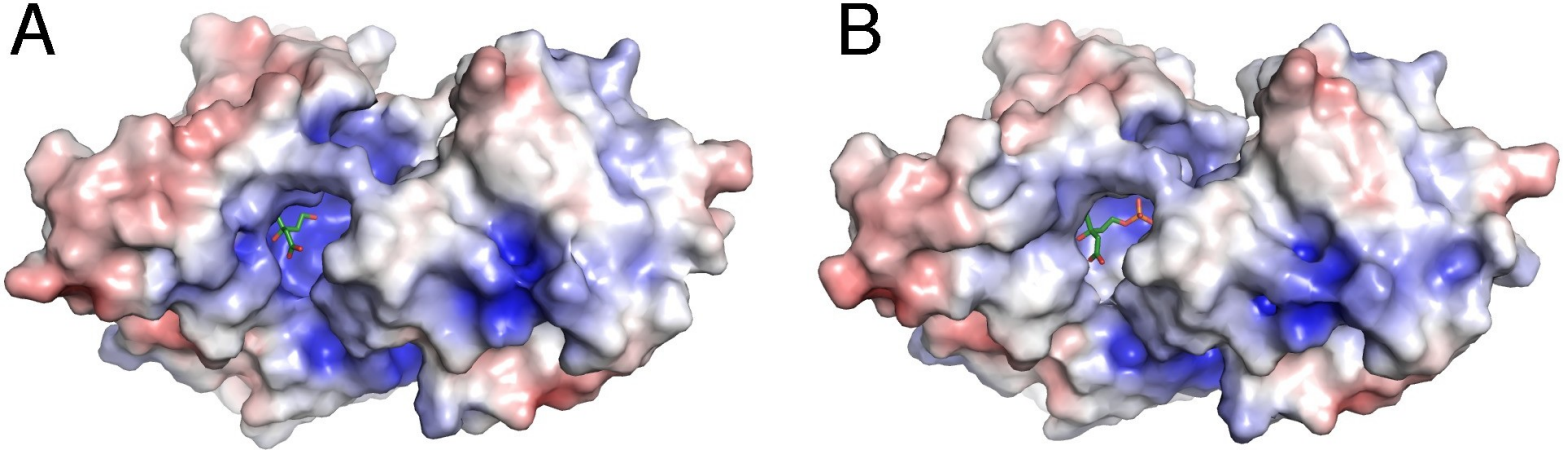
Electrostatic surface representation of substrate and product binding. (A) Mevalonate-bound and (B) 5-phosphomevalonate-bound MmMK shown in surface, with electrostatic potentials calculated using the Adaptive Poisson-Boltzmann Solver [31] in PyMOL [32], in which blue depicts greater positive charge, red depicts greater negative charge, and white depicts neutral regions. Mevalonate and 5-phosphomevalonate are shown as sticks.

Given the surprising lack of direct interactions between MmMK and mevalonate or 5-phosphomevalonate as well as any significant protein rearrangements, it is interesting to consider how substrate recognition might be achieved. It is possible that MmMK may simply provide a deep surface cleft of the appropriate size, geometry, and electrostatics to bind the substrate and the product (Fig 5A and 5B). Here, His16 is the crucial side chain for governing stereospecificity, as described above (Fig 4A–4C), and thus it follows that this residue provides the key interaction with both ligands.

### Mechanistic implications

**Fig 6:**
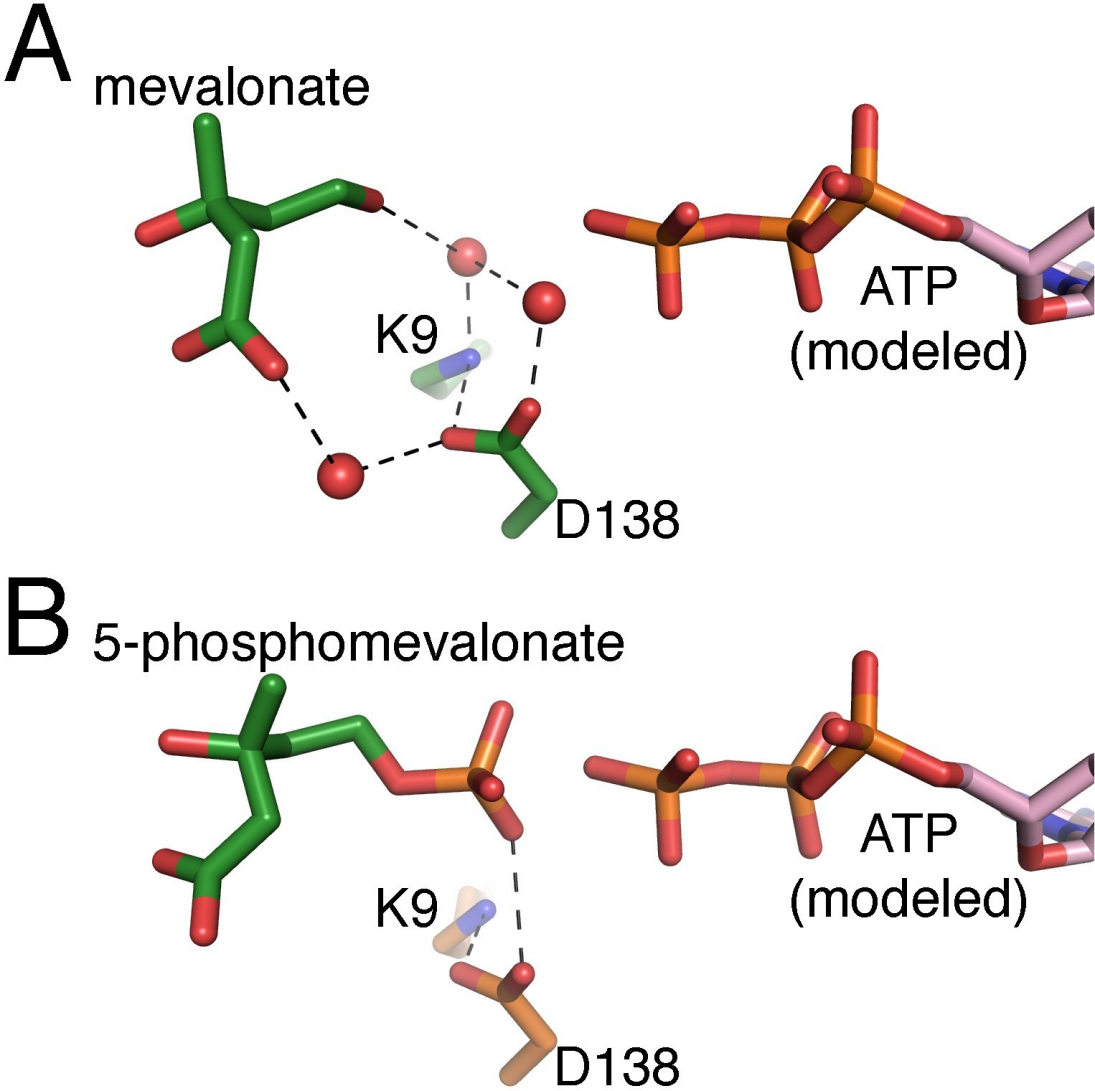
The MmMK active site. (A) The mevalonate-bound MmMK active site, with mevalonate and MmMK residues in green, and (B) the 5-phosphomevalonate-bound MmMK active site, with 5-phosphomevalonate in green and MmMK residues in orange. ATP modeled from an alignment with the ATP-bound RnMK structure (PDB entry 1KVK) is shown in pink.

These structures of MmMK bound with mevalonate and with 5-phosphomevalonate join the prior structure of unbound MmMK to provide a more complete portrait of the MK reaction, as apo-, substrate-bound, and product-bound structures of a single MK are now available. Although the structure ATP-bound MmMK has been elusive, the location of the ATP-binding site can be estimated by alignment with the structure of ATP-bound RnMK (Fig 6A and 6B), notwithstanding the fact that the specific ATP-binding regions and interactions are likely to be different between these two MK homologs. Therefore, though a full description of precisely how MmMK binds ATP awaits further structural characterization, our new MmMK structures presented here enable a deeper exploration of the MK mechanism.

**Fig 7:**
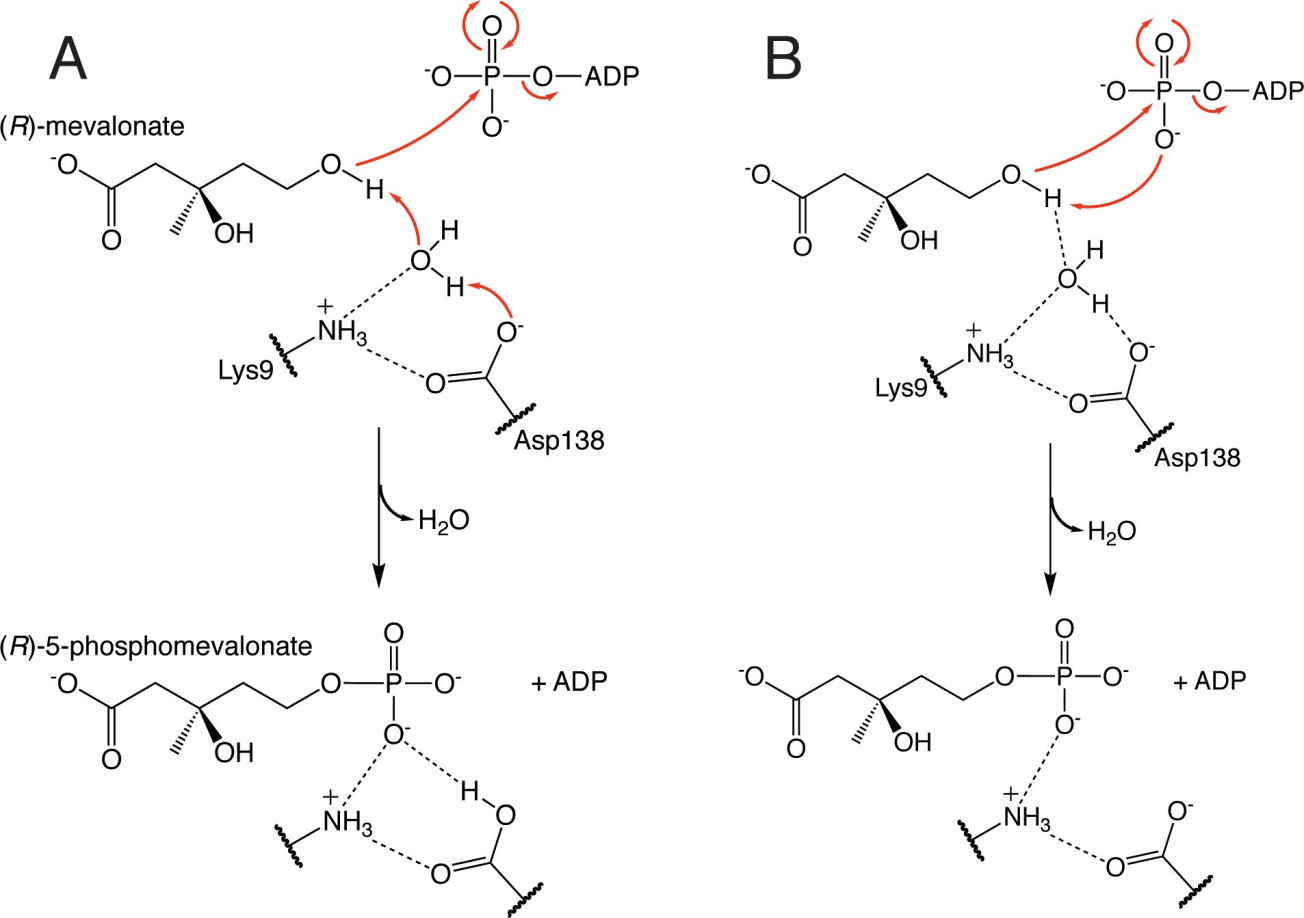
MK mechanistic proposals. (A) Proposed mechanism in which water molecules around mevalonate may deprotonate the C5-hydroxyl group of the substrate, assisted by conserved Asp138 acting as a base, to trigger phosphoryl transfer from ATP. (B) The alternative direct phosphorylation mechanism, in which the C5-hydroxyl group of mevalonate is deprotonated by the γ-phosphate group of ATP.

Once mevalonate binds the active site, its C5-hydroxyl group must be deprotonated to the active alkoxide nucleophile in order to attack the γ-phosphate of ATP. As mentioned above, mutagenesis studies indicate that conserved Asp138 performs this role. However, the prior mevalonate-bound LmMK structure as well as our mevalonate-bound MmMK structure presented here suggest that this aspartate residue may be too far from mevalonate to directly deprotonate the C5-hydroxyl, with distances of 5.2 and 5.5 Å, respectively. Instead, our structure contains ordered water molecules that bridge mevalonate and the protein including Asp138 (Fig 6A), suggesting that this residue is part of a hydrogen-bonding network involving water, which itself may directly deprotonate the substrate instead (Fig 7A). Still, mutation of this aspartate even to asparagine has a dramatic effect on activity while not significantly impacting substrate binding in human MK [15], suggesting a role in catalysis as a basic residue. Together, these data indicate that although water may deprotonate mevalonate, Asp138 is then also involved in mediating proton transfer from water, becoming protonated itself (Fig 7A). Our structure further shows that Lys9, strictly conserved among MKs, also participates in the hydrogen-bonding network with water (Fig 6A). However, this lysine residue is not thought to act as the catalytic base that deprotonates mevalonate, as detailed above. Instead, we observe Lys9 forming a salt bridge with Asp138 (Fig 7A), which may help to correctly orient Asp138 and ensure that Asp138 is in its deprotonated form upon mevalonate binding.

Another possibility is that the γ-phosphate group of ATP itself may deprotonate the C5-hydroxyl group of mevalonate to instigate phosphoryl transfer, a “direct phosphorylation” mechanism that is not reliant on any protein side chain acting as a general base (Fig 7B). Such a direct phosphorylation mechanism has been proposed in homoserine kinase (HSK), another GHMP kinase family member, as in HSK there is no ionizable side chain in the active site that is analogous to Asp138 of MK [33]. Lacking a protein residue in the active site that could serve as a base, HSK mechanistic proposals have instead depicted deprotonation by the ATP γ-phosphate. Indeed, in the crystal structure of the ternary HSK complex (PDB entry 1H72) that contains both homoserine and an ATP analog bound, only 3.4 Å separate the hydroxyl group of homoserine with the γ-phosphate of ATP [33]. In addition, molecular dynamics and computational studies with GHMP kinase family members galactokinase [34] and phosphomevalonate kinase [34] also suggested that the γ-phosphate could deprotonate the substrate, facilitated by stabilization afforded by nearby positively charged residues. Although it is possible that MK could also employ a similar direct phosphorylation mechanism, there is as yet no evidence for this mechanism in MK, and no structure analogous to the HSK structure has yet been determined of the ternary MK complex bound with both mevalonate and an ATP analog to depict both substrates poised for catalysis. Given the presence of strictly conserved Asp138 in MK and its demonstrated catalytic importance, currently available data instead suggest a catalytic role for Asp138, though the direct phosphorylation mechanism cannot be ruled out.

Regardless of the species that deprotonates the C5-hydroxyl group of mevalonate, the alkoxide species must attack the γ-phosphoryl group of ATP for phosphoryl transfer. Without a structure of the ternary MK complex, overlaying our structures with ATP modeled from the prior structure of ATP-bound RnMK [11] indicates that the alkoxide would be located directly in-line with the γ-phosphate of ATP (Fig 6A). Once the phosphoryl group is transferred to form 5-phosphomevalonate, our structure of product-bound MmMK depicts few structural alterations upon product formation (Fig 6B). Although we cannot assign protonation states of all ionizable groups, our structures suggest that the negatively charged phosphate group of the product now interacts with the now-protonated Asp138, consistent with its proposed role in deprotonation described above. Notably, fewer ordered water molecules appear to be bound in this region of our 5-phosphomevalonate MmMK structure (Fig 6B). Although the poorer resolution of the 5-phosphomevalonate-bound MmMK structure may itself hinder the visualization of water molecules, the presence of the phosphate group would necessarily displace water molecules that were present in this region of the mevalonate-bound structure. Together, these structures suggest that after water molecules deprotonate the C5-hydroxyl group of the mevalonate substrate, their positions become replaced by the new C5-phosphate group of the product and are released from the active site. With the product now formed, 5-phosphomevalonate may then leave the active site as well.

## Conclusions

With the X-ray crystal structures of archaeal MmMK bound with mevalonate and 5-phosphomevalonate presented here, structures are now available of apo, substrate-, and product-bound forms of a single MK for the first time. In particular, the structure of 5-phosphomevalonate represents the first crystal structure of any MK bound with its product. Unlike other MKs, MmMK also displays an unexpected lack of direct interactions with its substrate, due to a truncation of a β-strand, leaving the substrate more exposed to the solvent. We also identify a hydrogen-bonding network of ordered water molecules that mediates bridging interactions between catalytic residues of the protein and mevalonate and may be involved in substrate deprotonation to produce the active nucleophile for phosphoryl transfer to occur, yielding our product-bound structure. These structures and their mechanistic implications now provide a more complete view of MK activity and catalysis, representing a step forward in our understanding of a key reaction of the mevalonate pathway of steroid and isoprenoid biosynthesis.
